# Fusion of intra-oral scans in cone-beam computed tomography scans

**DOI:** 10.1007/s00784-020-03336-y

**Published:** 2020-06-03

**Authors:** F. Baan, R. Bruggink, J. Nijsink, T. J. J. Maal, E. M. Ongkosuwito

**Affiliations:** 1 Radboudumc 3DLab The Netherlands, Radboud university medical center, Radboud Institute for Health Sciences, Geert Grooteplein Zuid 10, 6525 GA Nijmegen, The Netherlands; 2 Department of Dentistry, section of Orthodontics and Craniofacial Biology, Radboud university medical center, Philips van Leydenlaan 25, 6525 EX Nijmegen, The Netherlands; 3 Department of Oral and Maxillofacial Surgery, Radboud university medical center, Geert Grooteplein Zuid 10, 6525 GA Nijmegen, The Netherlands; 4 Amalia Cleft and Craniofacial Centre, Radboud university medical centre, Geert Grooteplein Zuid 10, 6525 GA Nijmegen, The Netherlands

**Keywords:** Orthognathic surgery, Orthodontic(s), Oral implants/implantology, CAD, Treatment planning, Digital imaging/radiology

## Abstract

**Purpose:**

The purpose of this study was to evaluate the clinical accuracy of the fusion of intra-oral scans in cone-beam computed tomography (CBCT) scans using two commercially available software packages.

**Materials and methods:**

Ten dry human skulls were subjected to structured light scanning, CBCT scanning, and intra-oral scanning. Two commercially available software packages were used to perform fusion of the intra-oral scans in the CBCT scan to create an accurate virtual head model: IPS CaseDesigner® and OrthoAnalyzer™. The structured light scanner was used as a gold standard and was superimposed on the virtual head models, created by IPS CaseDesigner® and OrthoAnalyzer™, using an Iterative Closest Point algorithm. Differences between the positions of the intra-oral scans obtained with the software packages were recorded and expressed in six degrees of freedom as well as the inter- and intra-observer intra-class correlation coefficient.

**Results:**

The tested software packages, IPS CaseDesigner® and OrthoAnalyzer™, showed a high level of accuracy compared to the gold standard. The accuracy was calculated for all six degrees of freedom. It was noticeable that the accuracy in the cranial/caudal direction was the lowest for IPS CaseDesigner® and OrthoAnalyzer™ in both the maxilla and mandible. The inter- and intra-observer intra-class correlation coefficient showed a high level of agreement between the observers.

**Clinical relevance:**

IPS CaseDesigner® and OrthoAnalyzer™ are reliable software packages providing an accurate fusion of the intra-oral scan in the CBCT. Both software packages can be used as an accurate fusion tool of the intra-oral scan in the CBCT which provides an accurate basis for 3D virtual planning.

## Introduction

Three-dimensional (3D) virtual treatment planning is becoming an increasingly important tool within the fields of oral and maxillofacial surgery, orthodontics, and implantology [1–3]. Complex anatomical structures and the relations between these structures can be visualized using cone-beam computed tomography (CBCT). More specifically, bony structures and soft tissues can be captured using CBCT, which can be used to create a virtual head model [4, 5]. In addition, 3D stereophotogrammetry can be used to add texture and detail to the virtual head model [6]. To acquire accurate information about the dentition, additional imaging of the occlusal surfaces, e.g., intra-oral scans or plaster casts, is needed because of scattering present in a CBCT due to the high density of enamel, dental restorations, implants, and orthodontic appliances [7–9]. Accurately capturing the dentition is of major importance as drilling guides, saw guides, or orthognathic positioning guides are often dental occlusal-surface-supported [10–12]. Apart from accuracy, the dentition should also be positioned at the correct anatomical position in the mandible and maxilla. Therefore, accurate matching of the dentition in the CBCT is required for the use in clinical practice.

Several methods are described in literature to solve the issue of a distorted occlusal area within the CBCT scan. Swennen et al. [13] proposed a triple CBCT scan method using voxel-based registration to capture accurate occlusal surfaces. However, this requires the patient to be scanned twice, increasing the radiation exposure. Other methods have investigated the image-fusion of digital dental models with the CBCT using fiducial superimposition [14–17] or surface-based fusion [18, 19]. The study performed by Swennen et al. [13] is, to the best knowledge of the authors, the only study that utilized commercially available software.

The use of different imaging modalities (CBCT and plaster cast/intra-oral scans) allows surgeons and technicians to virtually plan and practice several treatments before the actual treatment takes place. Three-dimensional virtual orthognathic surgical planning is a widely used tool to plan and simulate different treatment options [3]. Additionally, in dental implantology, digital implant planning is also widely used to preoperatively assess the bone quality and digitally plan the optimal implant position. Three-dimensional printed surgical drilling guides can be used to transfer the digital plan towards the operating theater [20]. Within the orthodontic work field, 3D techniques are used for several different applications. Orthodontic virtual setups can be created and used to assess the accuracy of the treatment. [21] Digitally created indirect bonding trays can be used for optimal bracket placement and an enhancement of the workflow [22].

In the current workflow, taking physical impressions, and pouring them into plaster casts, is the most used technique to capture the maxillary and mandibular occlusal surfaces precisely [23–28]. With the introduction and technical evolution of intra-oral scanners, it became easier and quicker to obtain a detailed model of the dentition of the patient without the need for physical impressions. Earlier studies proved that an intra-oral scanner is a valid method for accurately visualizing the dentition. Furthermore, patients generally experience an intra-oral scan as a more comfortable way of getting an accurate 3D dentition compared to physical impressions [28–31].

The purpose of this study is to assess the accuracy of the replacement of distorted dentition in CBCT scans with accurate digital dental models, using commercially available software.

## Materials and methods

Ten dry human cadaveric skulls were obtained from the historical archive of the Radboud Anatomical Museum. For this study, no approval was required from an ethical committee. The selected cadaveric skulls had intact bony structures, ≥ 24 teeth, and ≤ 10 dental restorations. Dental status was recorded and summarized in Table 1.

**Table 1:** Dental status of the included cadaveric skulls. FDI annotation is used for tooth numbering

Skull no.	Dental range	Missing tooth	Filled tooth	Dental range	Missing tooth	Filled tooth
	Maxilla			Mandible		
1	17–27	–	16, 17, 26	37–47	36, 32, 46	34, 47
2	17–28	13, 25	–	38–48	–	–
3	17–27	–	–	37–47	–	–
4	18–28	14, 17, 25	–	38–48	–	–
5	18–28	–	15, 27	38–48	–	37, 46
6	18–28	–	16	38–48	–	36, 46
7	17–27	–	24, 26	37–47	–	33, 45
8	17–27	16, 23	15, 17	36–47	46	35
9	18–28	–	–	38–48	–	–
10	18–28	–	–	38–48	–	–

### Data acquisition

#### Study data

The ten dry skulls were scanned with a KaVo 3D Exam CBCT scanner (KaVo Germany) with an extended height protocol (FOV 23 × 17 cm at 120 kV and 0.4 mm isotropic voxel size). The skulls were scanned with a wax bite in place to ensure a centric relation. After CBCT scanning, the dentition was scanned using an intra-oral scanner (TRIOS3, 3shape A/S, Copenhagen, Denmark). The maxillary and mandibular dentition were scanned separately and exported in the Standard Tessellation Language (STL) format.

#### Gold standard

All ten skulls were also digitized using a structured light scanner (Scan in a box-FX, Open Technologies Srl, Italy) with an accuracy of 100 μm as specified by the manufacturer. The 3D surface models were exported in the STL format. The 3D surface models contain detailed information of the bony parts of the skull as well as detailed information of the dentition and were therefore used as the gold standard in this study.

#### Fusion

**Fig. 1:**
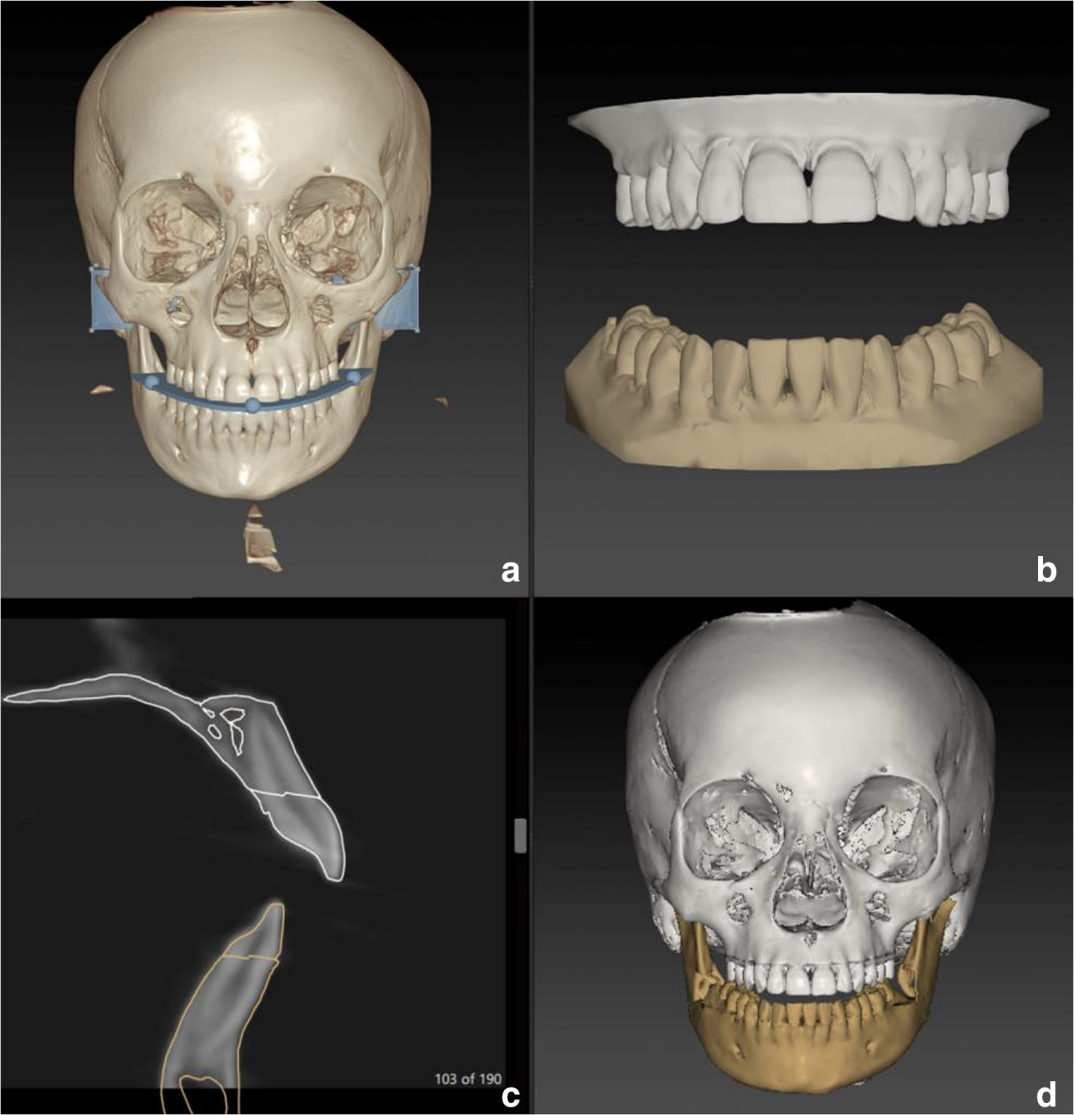
Overview of the fusion process in the IPS CaseDesigner®. **a** Three-dimensional CBCT skull model in the IPS CaseDesigner® software with the left and the right condyle indicated as well as the occlusion plane. **b** Intra-oral scans of maxilla and mandible. **c** Cross-sectional view of the 3D CBCT skull model and fused intra-oral scans. **d** Frontal view of the result of the fusion of the intra-oral scans in the CBCT

The fusion of the dentition from the intra-oral scans in the CBCT scans was studied for two different commercial software packages. The first software package tested in this study was IPS CaseDesigner® (KLS Martin, Tuttlingen, Germany). To ensure proper fusion, the STL models of the dental arches created with the intra-oral scanner were rotated 90° around the *x*-axis to align them with the coordinate system of IPS CaseDesigner®. Rotating the STL files was needed because the coordinate system used by the intra-oral scanner was not the same as the coordinate system of IPS CaseDesigner®. The DICOM files of the extended height CBCT scan were imported to create a 3D virtual head model. The rotated STL models of the dental arches created were also imported. By indicating the right and left condyle, mesial cusp of the first upper right and left molar and the middle of the two upper incisors, IPS CaseDesigner® automatically aligns the STL models of the dentition with the 3D virtual head based on contrast information at the tooth crown margin (Fig. 1). After alignment, no manual adjustment was performed and the virtual head model with the incorporated intra-oral scans were exported in their correct position.

**Fig. 2:**
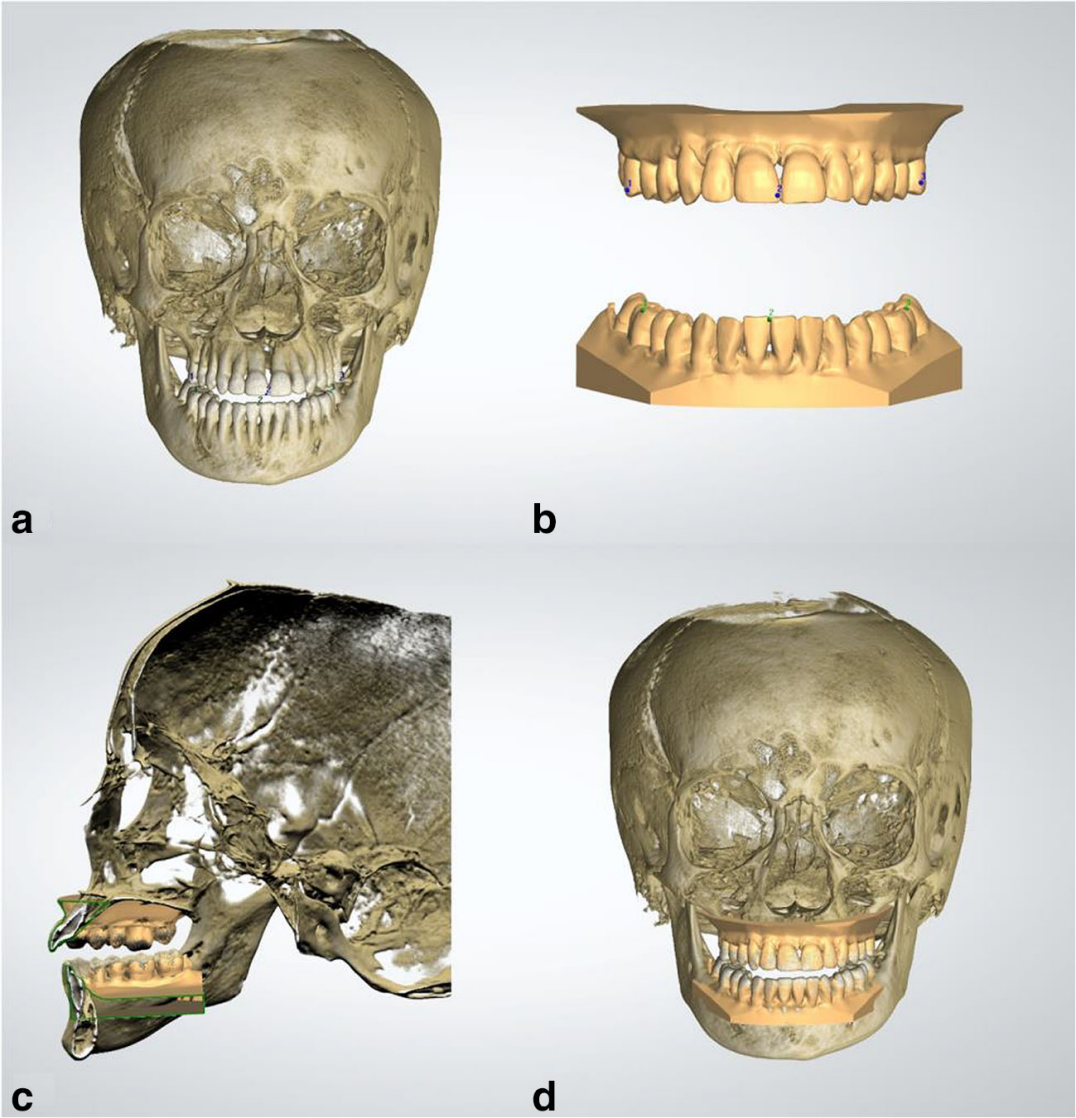
Overview of the fusion process of the intra-oral scans in the CBCT in OrthoAnalyzer™. **a** Three-dimensional CBCT skull model in OrthoAnalyzer™ with three points on both the maxillary and mandibular teeth. **b** Intra-oral scans of maxilla and mandible with three points on both the maxillary and mandibular teeth, corresponding with the points shown in 3A. **c** Cross-sectional view of the 3D CBCT skull model and fused intra-oral scans (shown in green). **d** Frontal view of the result of the fusion of the intra-oral scans in the CBCT

The second software package tested was the OrthoAnalyzer™ software (3shape A/S, v2019, Copenhagen, Denmark). The DICOM files of the extended height CBCT scan were imported together with the STL models of the dentition. The STL models of the dental arches were not rotated as the coordinate system between the intra-oral scanner and the OrthoAnalyzer™ software was the same. Two sets of corresponding dental landmarks (frontal incisor, left and right molar) were selected on both the 3D CBCT head model and intra-oral scans (Fig. 2). First, OrthoAnalyzer™ roughly aligned the skull model and the intra-oral scans based on the two sets of corresponding landmarks. Second and last, the shape and outline of the dentition was used for automatic alignment. No manual adjustment was performed after aligning the intra-oral scans with the CBCT model and the virtual head model intra-oral scans were exported in their new position.

#### Accuracy study

**Fig. 3:**
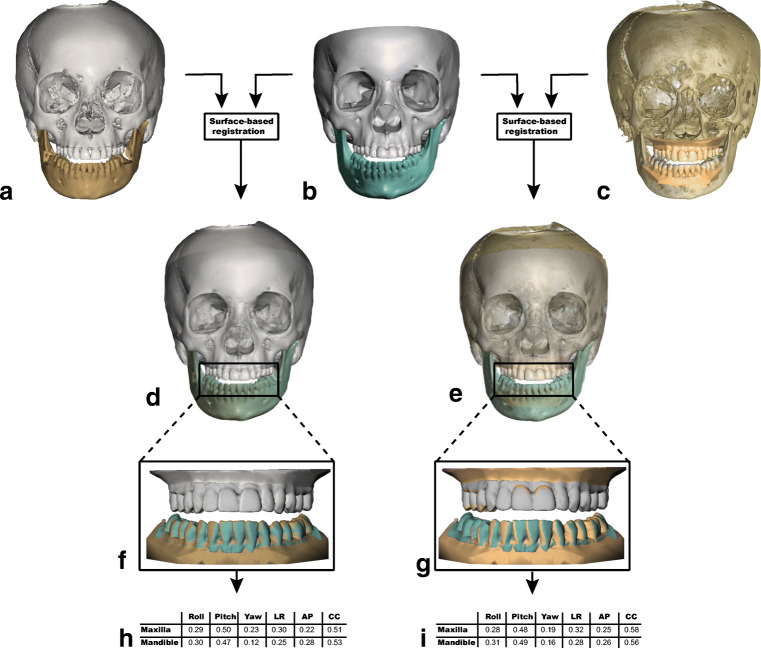
Overview of the fusion process. (A) Virtual head model made with IPS CaseDesigner®. (B) Structured light scan (gold standard) of a dry humal skull with high detail of the bony structures as well as the teeth. (C) Virtual head model made with OrthoAnalyzer™. (D) Superimposition of the gold standard with the virtual head model created in IPS CaseDesigner® using the forehead, orbital rims, and zygomatic area as reference. (E) Superimposition of the gold standard with the virtual head model created in OrthoAnalyzer™ using the forehead, orbital rims, and zygomatic area as reference. (F) The different positions of the dental arches of IPS CaseDesigner® and the gold standard. Blue and gray: dental arch position of the gold standard, brown and white: position of the dental arches fused using IPS CaseDesigner®. (G) The different positions of the dental arches of OrthoAnalyzer™ and the gold standard. Blue and gray: dental arch position of the gold standard, brown: position of the dental arches fused using IPS CaseDesigner®. (H) Differences of the dental arches expressed in six DOF for IPS CaseDesigner® and the gold standard for both the maxilla and mandible. (I) Differences of the dental arches expressed in six DOF for OrthoAnalyzer™ and the gold standard for both the maxilla and mandible. LR left/right, AP anterior/posterior, CC cranial/caudal

To investigate the accuracy of the fusion of the intra-oral scan with the extended height CBCT scan, the first step was to align the structured light scan of the skull with the virtual head model created with IPS CaseDesigner® (Fig. 3a) and OrthoAnalyzer™ (Fig. 3c). The virtual head models created with IPS CaseDesigner® and OrthoAnalyzer™ were imported into 3DMedX (v1.2.0.4, 3D Lab Radboudumc, Nijmegen) as well as the structured light scan (Fig. 3c). An iterative closest point (ICP) algorithm [32] was used to align the different head models from the different software packages. ICP registration is an accurate and reliable method for registration of similar surfaces [32]. The algorithm enables the alignment of different 3D surfaces in such a way that the difference between the two surfaces is minimized. The intra-oral scans were incorporated in the virtual head models. The ICP algorithm was used to align the structured light scan towards the virtual head models created with IPS CaseDesigner® and OrthoAnalyzer™ using the forehead, orbital rims and zygomatic area as reference (Fig. 3c, d). Distance maps were calculated to assess the accuracy of the alignment of the virtual head models with the structured light scan (gold standard).

Finally, the intra-oral scans from the OrthoAnalyzer™ and IPS CaseDesigner® were displaced towards the structured light scanned dentition using the ICP algorithm (Fig. 3b). The ICP algorithm aligns the different surfaces and also provides a transformation matrix of the exact displacement needed to perfectly align the intra-oral scans from the OrthoAnalyzer™ and IPS CaseDesigner® towards the structured light scanned dentition. The transformation matrix contains the translational and rotational directions. The transformation matrix was converted to clinically relevant information using the same method as described by LaValle [33]. The translations and rotations were recorded and converted to the six degrees of freedom (DOF) for the dentition of the maxilla and the dentition of the mandible (Fig. 3h, i).

#### Statistics

Data was tested for normality using the Shapiro-Wilk test for small sample size. A paired sample *t* test was used to evaluate the accuracy of the placement of the intra-oral scan in the CBCT for both IPS CaseDesigner® and OrthoAnalyzer™. To assess the reproducibility of the fusion, two experienced observers repeated the fusion in both the IPS CaseDesigner® and the OrthoAnalyzer™ software for all ten dry skulls. The intra- and inter-observer reproducibility of the fusion for OrthoAnalyzer™ and IPS CaseDesigner® were assessed using the intraclass correlation coefficient. For all tests, the significance level was set at *p* < 0.05.

## Results

A total of ten dry skulls were used in the study. All skulls had ≥ 24 teeth in order to achieve a proper fusion of the intra-oral scan in the CBCT. Data distribution was tested for normality using the Shapiro-Wilk test and showed a normal distribution (*p* > 0.05).

### Gold standard

Aligning the structured light scan (gold standard) towards the CBCT using the ICP algorithm in the 3DMedX software, using the forehead, orbital rims and zygomatic area as reference, resulted in an average matching accuracy of 0.20 ± 0.16 mm for the skull/maxilla and 0.11 ± 0.12 mm for the mandible.

### Accuracy

The translational directions investigated were left/right, anterior/posterior, and cranial/caudal. For the rotational directions, the Pitch (rotation around the *x*-axis), Roll (rotation around *y*-axis), and Yaw (rotation around *z*-axis) rotations were used. Translational and rotational directions are displayed in Table 2 for both IPS CaseDesigner® and OrthoAnalyzer™. Corresponding *p* values are also given in Table 1 as well as the *p* values for the statistical differences between IPS CaseDesigner® and OrthoAnalyzer™.

**Table 2:** Accuracy of the fusion of the intra-oral scan with the CBCT compared to the gold standard expressed in the translational directions *x* (left-right), *y* (anterior-posterior), *z* (cranial-caudal), overall accuracy and rotational directions Pitch (rotation around *x*-axis), Roll (rotation around *y*-axis), and Yaw (rotation around *z*-axis)

		LR (mm) Mean ± SD *p* value*	AP (mm) Mean ± SD *p* value*	CC (mm) Mean ± SD *p* value*	Overall difference (mm)	Pitch (°) Mean ± SD *p* value*	Roll (°) Mean ± SD *p* value*	Yaw (°) Mean ± SD *p* value*
IPS Case Designer®	Maxilla	0.29 ± 0.19 0.01	0.25 ± 0.31 0.01	0.40 ± 0.28 0.02	0.60	0.25 ± 0.20 0.01	0.29 ± 0.28 < 0.01	0.18 ± 0.13 < 0.01
	Mandible	0.30 ± 0.31 0.01	0.36 ± 0.40 0.01	0.45 ± 0.46 < 0.01	0.66	0.32 ± 0.24 < 0.01	0.22 ± 0.20 0.01	0.16 ± 0.12 < 0.01
Ortho-Analyzer™	Maxilla	0.47 ± 0.29 0.00	0.62 ± 0.53 0.00	0.83 ± 0.54 0.00	1.18	0.26 ± 0.20 0.00	0.45 ± 0.35 0.00	0.28 ± 0.29 0.01
	Mandible	0.41 ± 0.44 < 0.01	0.62 ± 0.49 < 0.01	0.78 ± 0.76 0.01	1.09	0.37 ± 0.47 0.02	0.21 ± 0.21 0.01	0.23 ± 0.22 < 0.01
*p* value**	Maxilla	0.65	0.06	0.26	–	0.71	0.04	0.34
	Mandible	0.01	0.08	0.18	–	0.85	0.57	0.13
		0.19	0.37	0.43	–	0.01	0.16	0.10
		0.11	0.28	0.33	–	0.05	0.01	0.07

Overall difference is defined as the 3D distance between the commercial software and the gold standard

*LR* left/right, *AP* anterior/posterior, *CC* cranial/caudal, *SD* standard deviation

*p value of the difference between the commercial software and the gold standard, **p value of the difference between IPS CaseDesigner® and OrthoAnalyzer™; the significance level was set at *p* < 0.05

For IPS CaseDesigner®, translational differences compared to the gold standard were all smaller than the voxel size of the CBCT (0.40 mm) except for the cranial/caudal direction which was the largest in the mandible (0.45 ± 0.46 mm). Rotational differences compared to the gold standard did not exceed 0.32**°** with the yaw of mandible showing the smallest difference (0.16 ± 0.12°) and the roll of the maxilla the largest (0.29 ± 0.28°). The paired *t* test showed a statistically significant difference between the gold standard and IPS CaseDesigner® for all translations and rotations. However, the differences were ≤ 0.40 mm except for the cranial-caudal translation.

For the OrthoAnalyzer™ software, the translational differences were the largest for cranial/caudal direction of the maxilla (0.83 ± 0.54 mm) whereas the left/right direction of the mandible showed the smallest inaccuracy (0.41 ± 0.44 mm). Rotational differences did not exceed 0.45°, with the roll of the mandible showing the smallest difference (0.21 ± 0.21°) and the roll of the maxilla the largest (0.45 ± 0.35°). Translational and rotational differences all statistically differ from the gold standard for the OrthoAnalyzer™.

IPS CaseDesigner® showed the smallest overall difference for the maxilla (0.60 mm) whereas the maxilla of the OrthoAnalyzer™ showed the largest difference (1.18 mm).

Comparing both software packages, OrthoAnalyzer™ showed bigger translational discrepancies compared to IPS CaseDesigner®. Only the left/right direction of the mandible (*p* = 0.01) showed a statistically significant difference. Rotational differences were all smaller than 0.50° for all ten skulls, and only the Roll of the maxilla showed a statistically significant difference (*p* = 0.04).

### Reproducibility

The intra-observer overall mean difference was 0.08 ± 0.10 mm for IPS CaseDesigner® and 0.18 ± 0.15 mm for OrthoAnalyzer™. The inter-observer overall mean difference was 0.11 ± 0.12 mm for IPS CaseDesigner® and 0.21 ± 0.19 mm for OrthoAnalyzer™. The intra- and inter-observer reliability analysis showed good correlation and intraclass coefficients for OrthoAnalyzer™. High correlation and intraclass coefficients were found for IPS CaseDesigner®. In comparison with IPS CaseDesigner®, OrthoAnalyzer™ showed lower scores. The results of the analysis are shown in Table 3.

**Table 3:** The intraclass correlation coefficient displayed for all translations and rotations for both the maxilla and mandible

			LR	AP	CC	Roll	Pitch	Yaw
IPS Case Designer®	Maxilla	Intra-observer ICC	0.97	0.98	0.97	0.93	0.95	0.97
		Inter-observer ICC	0.94	0.98	0.92	0.92	0.97	0.90
	Mandible	Intra-observer ICC	0.99	0.99	0.98	0.99	0.98	0.97
		Inter-observer ICC	0.97	0.97	0.93	0.99	0.99	0.96
Ortho Analyzer™	Maxilla	Intra-observer ICC	0.90	0.84	0.89	0.89	0.83	0.92
		Inter-observer ICC	0.84	0.85	0.82	0.88	0.88	0.90
	Mandible	Intra-observer ICC	0.89	089	0.92	0.90	0.92	0.93
		Inter-observer ICC	0.88	0.84	0.89	0.90	0.90	0.93

*ICC* intraclass correlation coefficient

## Discussion

CBCT imaging is a widely used tool for capturing the human skull. However, CBCT has the drawback that it is prone to distortions around the dentition. Metallic restorations, orthodontic appliances, and the high density of enamel cause distortion of the dentition in the CBCT model [7–9]. In order to utilize CBCT imaging for CAD/CAM processes, additional imaging is needed as well as a proper fusion between the different imaging modalities. In a recent review of Mangano et al. [34], it was concluded that there was still no easy way to fuse scans from different image modalities.

In this study, the accuracy of the fusion of intra-oral scans into CBCT models was assessed using commercially available software. The tested software packages, IPS CaseDesigner® and OrthoAnalyzer™, showed a high level of accuracy compared to the gold standard. The accuracy was calculated for all six degrees of freedom. It was noticeable that the accuracy in the cranial/caudal direction was the lowest for IPS CaseDesigner® and OrthoAnalyzer™ in both the maxilla and mandible. A logical reason for this lower accuracy could not be found, but it is worth to note this difference. The user of these software packages should take this larger inaccuracy in the cranial/caudal direction into account when performing a surgical planning. A visual check whether the software performed an accurate fusion is strongly advisable.

An important step in assessing the fusion accuracy is the alignment of the structured light scan of the skull (gold standard) with the CBCT 3D model. This was performed utilizing a validated ICP algorithm [32]. The accuracy of the alignment of the gold standard with the CBCT 3D model was 0.20 mm. This is a clinically acceptable result as the resolution of the CBCT scans was 0.40 mm.

Overall mean intra- and inter-observer differences were low (≤ 0.21 mm) which was reflected in the ICC values found. In the OrthoAnalyzer™, the user needs to provide three corresponding points on both the CBCT 3D model and the intra-oral scan. As the results show, this increases the intra- or inter-observer variability in OrthoAnalyzer™ compared to the workflow in IPS CaseDesigner which did not use additional manual input. However, all ICC values were > 0.82 showing good agreement for OrthoAnalyzer™ and > 0.92 for IPS CaseDesigner® showing excellent intra- and inter-observer agreement.

When comparing the fusion techniques of this current study to earlier studies, it is noteworthy that all earlier studies either utilize intra-oral markers [17, 35, 36], extra-oral markers [15, 37], or utilized a double/triple scanning procedure [13]. The fusion technique utilized by IPS CaseDesigner® and OrthoAnalyzer™ does not require markers or an additional CBCT scan. This makes it a convenient method for use in the clinical practice. Moreover, the accuracy found for IPS CaseDesigner® is in line with other studies. A splint with ceramic balls was used by Uechi et al. [15], and a root-mean-square error of 0.4 mm was found. Another study found an accuracy ranging from 0.10 to 0.50 mm by using titanium markers [37]. De Waard et al. found errors ranging from 0.12 to 0.45 mm. Another study performed by Lin et al. using surface-based matching found errors ranging from 0.11 to 0.53 mm. However, most of these studies assessed the accuracy of the fusion (e.g., how do the markers overlap) instead of assessing the differences using a true gold standard. The accuracies found for IPS CaseDesigner are in the range of these studies as the overall accuracy is ≤ 0.66 mm. OrthoAnalyzer shows bigger discrepancies (≤ 1.18 mm) and is therefore not in line with earlier studies.

A limitation of the current study is the use of dry human skulls which could influence the result of the study. For example, accuracy of intra-oral scanning can be lower in actual patients as patient movement, anatomical restrictions, and excessive saliva can hamper proper imaging of the dentition [28]. Another limitation of the current study was the absence of orthodontic appliances. Most orthognathic patients have orthodontic brackets which can influence the accuracy of the fusion as might cause distortion of the CBCT scan. Therefore, a future study to investigate the effects of orthodontic appliances is necessary. Furthermore, to study the use of the fusion of intra-oral scans in CBCT in implantology patients closer, a study should be designed in which partial dental arches are used to see whether the fusion is still accurate if more teeth are missing.

Recent developments in artificial intelligence (AI) are promising. Earlier studies showed that using AI, it is possible to segment third molars from an orthopantomogram [38]. With future developments, AI might be a promising technique to automatically “recognize” dentition in a CBCT image. Recognizing the dentition might make it easier to replace it and therefore enhancing the accuracy of the fusion between intra-oral scans and CBCT. As AI (e.g., convolutional neural networks) is an upcoming and promising technique in image fusion [39], development of AI-driven algorithms to fuse dental information with CBCT data might result in a more accurate and automated solution.

## Conclusion

IPS CaseDesigner® is a reliable software packages within the scope of this study. It provides accurate fusion of the intra-oral scan in the CBCT when a complete dental arch is used with little to none dental fillings. OrthoAnalyzer™ showed bigger discrepancies, and therefore, it is recommended to perform proper visual inspection before using the fusion. Inaccuracies were found in both packages. However, for IPS CaseDesigner, these are in line with the findings of similar studies. OrthoAnalyzer shows bigger discrepancies. Future research towards the effect of scattering caused by fillings and orthodontic appliances is recommended as well as the influence of missing teeth.
